# Isoliquiritigenin Elicits Potent Antiviral Activity Against Pseudorabies Virus Through Modulating the cGAS/STING and JAK/STAT Signaling Cascades

**DOI:** 10.3390/vetsci13090974

**Published:** 2026-09-16

**Authors:** Hongqiao Hu, Ying Yang, Jinbin Zhang, Yican Wang, Xun Zhou, Lixia Li, Yuanfeng Zou, Mingyue Li, Shujun Dai, Yifei Lang, Xu Song

**Affiliations:** 1 Natural Medicine Research Center, College of Veterinary Medicine, Sichuan Agricultural University, Chengdu 611130, China; 17764812036@163.com (H.H.); yyyang202608@163.com (Y.Y.); ast_952@163.com (J.Z.); 18782339807@163.com (Y.W.); zhouxun0212@outlook.com (X.Z.); lilixia905@163.com (L.L.); yuanfengzou@sicau.edu.cn (Y.Z.); 17321905677@163.com (M.L.); 2 Ningxia Guiliu Benniu AB Agri Biotechnology Co., Ltd., Yinchuan 750000, China; shujundai@126.com

**Keywords:** antiviral activity, isoliquiritigenin, pseudorabies virus, type I interferon responses

## Abstract

Pseudorabies virus (PRV), a member of the *Alphaherpesvirinae* subfamily, poses a significant economic threat to the global swine industry and has raised growing public health concerns due to its cross-species transmission potential. Given emerging variants and the fact that current vaccines are restricted to pigs, novel antiviral agents are urgently needed. The present study assessed the anti-PRV activity of isoliquiritigenin, as well as its modulatory role in innate immune pathways, using cell-based assays and a PRV-infected mouse model. The results showed that isoliquiritigenin exhibited a half-maximal inhibitory concentration (IC_50_) of 35.47 μM and a selective index of 3.34 in PK-15 cells, with efficacy sustained at high infectious dose. Mechanistically, it counteracted PRV-induced suppression of type I interferon responses, correlating with core genes in the cGAS/STING pathway and enhancing IRF3 phosphorylation, while also activating the JAK/STAT axis and increasing STAT1 phosphorylation. In vivo, treatment with isoliquiritigenin decreased mortality by 40 percentage points (from 70% to 30%), substantially suppressed viral burden in various tissues, and concomitantly activated type I interferon signaling. Collectively, these findings suggest that isoliquiritigenin potentiates host innate immunity, potentially through the cGAS/STING and JAK/STAT cascades, offering a novel flavonoid-based lead for anti-herpesvirus drug development.

## Simple Summary

Pseudorabies virus infects pigs and can also spread to other animals and humans. Current vaccines are only for pigs and cannot control new viral variants, so new treatments are urgently needed. A natural compound from licorice, isoliquiritigenin, was demonstrated to block viral replication, even when challenged with high multiplicities of infection. Rather than attacking the virus directly, this compound boosts the body’s natural immune defenses. In the infected mice, it resulted in a 40-percentage-point reduction in mortality (from 70% to 30%) and significantly lowered viral loads in multiple organs. This study suggests that isoliquiritigenin could become a natural therapy to protect the pork industry against pseudorabies virus infection.

## 1. Introduction

PRV, also known as porcine herpesvirus type 1 (PHV1), is classified as an α-herpesvirus, specifically within the Varicellovirus genus [1,2]. Swine serve as the natural hosts, and PRV can also infect a broad spectrum of other animals, including ruminants, carnivores, rodents, and lagomorphs [3]. Infection with PRV results in considerable economic losses for the global livestock industry, particularly in swine farming. Pseudorabies has been shown to cause nearly 100% mortality in piglets, reproductive failure in sows, growth retardation in finishing pigs, and increased susceptibility to secondary infections. Although eradication programs can enhance farm profitability, they entail significant costs [4].

The mainstay of PRV control is vaccination; however, currently available vaccines are licensed exclusively for use in pigs and face dual challenges related to efficacy and safety. Since 2011, outbreaks in Bartha-K61-vaccinated herds in China have persisted due to emerging variant strains [5,6]. These variants exhibit significant genetic and antigenic divergence from classical strains, with vaccine efficacy declining from 100% against the classical SC strain to just 50% [7]. The 2022 isolate CH/JLHF/2022 largely evaded maternal antibodies, causing 100% mortality in piglets [8]. Adding to these efficacy concerns, a 2025 case report raised a potential safety issue: Chen et al. [9] reported an outbreak in PRV-negative Danish purebred pigs imported to Taiwan, China. Both groups exhibited 100% morbidity, with mortality rates of 67.6% and 13.4%, accompanied by severe immunopathology. These observations suggest a possible association with Bucharest strain live vaccine (Zoetis PR-VAC PLUS) immunization, constituting a potential safety signal. As a potential zoonotic pathogen, PRV has caused markedly rising human infections, manifesting as viral encephalitis [10,11,12] and endophthalmitis [13], the latter often leading to permanent blindness [2,14]. This highlights its growing threat to public health, particularly to swine industry workers [15].

Type I interferons (IFN-α/β) are key mediators of the innate antiviral response. Their production is initiated upon host recognition of viral nucleic acids by pattern recognition receptors (PRRs) such as Toll-like receptors (TLRs) and RIG-I-like receptors (RLRs), as well as the DNA sensor cGAS. This recognition activates the cGAS/STING pathway, which triggers downstream cascades involving TBK1 kinase and leads to phosphorylation of the transcription factors IRF3 and IRF7. Phosphorylated IRF3/7 then dimerize and translocate to the nucleus to drive type I interferon gene expression [16]. Once secreted, type I IFNs bind to the IFN-α/β receptor (IFNAR), activating the JAK-STAT pathway and leading to the formation of the ISGF3 complex (STAT1-STAT2-IRF9). ISGF3 translocates to the nucleus to induce the expression of numerous interferon-stimulated genes (ISGs). The resulting ISG products inhibit viral replication through diverse mechanisms, such as blocking protein translation, degrading viral nucleic acids, or modulating cellular metabolism, thereby establishing an antiviral state at the site of infection [17].

Flavonoids, a class of naturally occurring polyphenolic compounds, have been demonstrated to exert regulatory effects on specific phases of the viral life cycle, thereby impeding viral replication in diverse viruses, including herpesviruses [18]. Wogonin has been shown to modulate NF-κB and JNK/p38 MAPK pathways to exert anti-HSV activity [19]. Similarly, naringenin has been shown to exert antiviral activity, with effects including inhibition of virion assembly and modulation of viral protein translation [20]. Among these bioactive flavonoids, isoliquiritigenin, a hydroxychalcone derived from licorice root, has attracted considerable interest due to its pleiotropic pharmacological effects, including antioxidant, anti-inflammatory, antidiabetic, cardioprotective, hepatoprotective, neuroprotective, and anticancer properties [21]. To date, studies on the antiviral activity of isoliquiritigenin remain limited. A preliminary in vitro study has reported that isoliquiritigenin can reduce the viral titers of vesicular stomatitis virus, influenza A virus, encephalomyocarditis virus, and herpes simplex virus type 1 [22]. Additionally, isoliquiritigenin exhibits potent antiviral activity against spring viremia of carp virus (SVCV) in aquaculture models, an effect attributed to the dual modulation of host antioxidant defenses and immune responses [23]. However, its efficacy against PRV and the underlying mechanisms remain unexplored. Given the critical role of IFN-I in controlling PRV infection and the virus’s ability to subvert this pathway, this study aimed to evaluate the regulatory effects of isoliquiritigenin against PRV both in vitro and in vivo, focusing on the cGAS/STING and JAK/STAT signaling cascades, to clarify its antiviral mechanism and facilitate the development of novel anti-PRV agents.

## 2. Materials and Methods

### 2.1. Cell, Virus, and Isoliquiritigenin

The porcine kidney cell line (PK-15) and African green monkey kidney cells (Vero) were obtained from the China Center for Type Culture Collection (China Center for Type Culture Collection, Wuhan, China). Both cell lines were cultured in Eagle‘s Minimum Essential Medium (EMEM) supplemented with 10% (*v*/*v*) calf serum (Gibco, Grand Island, NY, USA), 100 U/mL penicillin, and 100 μg/mL streptomycin. For the maintenance medium (MM), the serum concentration was reduced to 2%.

The pseudorabies virus (Ra strain), a virulent field isolate, was purchased from the China Veterinary Culture Collection Center (Beijing, China) and propagated in PK-15 cells. After five passages, the virus was maintained at the Natural Medicine Research Center, Sichuan Agricultural University (Chengdu, China). Prior to use in this study, the virus was passaged for an additional 10 passages in PK-15 cells. The 50% tissue culture infectious dose (TCID_50_) of the virus stock was determined to be 10^5.7^TCID_50_/mL according to the Reed–Muench method.

Isoliquiritigenin (catalog No. Y91617A, Adamas Life, manufacturer direct, Shanghai, China) with a purity of ≥97% (determined by actual test; Bio-Active Grade) was used in this study. The compound was dissolved in dimethyl sulfoxide (DMSO; Beijing Solarbio Science & Technology Co., Ltd., Beijing, China) to prepare a 200× stock solution, which was then diluted with maintenance medium to the desired working concentrations immediately before use. The final DMSO concentration in all experimental groups was ≤0.5%.

### 2.2. Cytotoxic Concentration Assay

The cytotoxicity of isoliquiritigenin against PK-15 cells was evaluated using the CCK-8 assay. PK-15 cells were seeded in 96-well plates and treated with two-fold serial dilutions of isoliquiritigenin ranging from 1000 to 15.63 µM, with six replicates per concentration. After incubation for 48 h, 10 µL of CCK-8 solution was added to each well, and the plates were further incubated at 37 °C for 30 min. Absorbance was then measured at 450 nm using a microplate reader. Cell viability was calculated as: (OD_ISL_ − OD_blank_)/(OD_control_ − OD_blank_) × 100%, where OD_ISL_ represents cells treated with isoliquiritigenin, OD_control_ represents untreated cells, and OD_blank_ represents medium-only wells. The half-maximal cytotoxic concentration (CC_50_) was determined by fitting the dose–response data to a nonlinear regression model (log(inhibitor) vs. normalized response, variable slope) using GraphPad Prism 9.0 (Dotmatics, Boston, MA, USA).

### 2.3. Antiviral Activity Assay

PK-15 cell monolayers in 96-well plates were infected with PRV at 100 TCID_50_ per well. For this, the virus stock with a titer of 10^5^·^7^ TCID_50_/100 µL was diluted 10^−3.7^ (approximately 5000-fold) in maintenance medium to obtain 100 TCID_50_ per 100 µL, and 100 µL of this dilution was added to each well. One hour post-infection, the virus-containing medium was removed and replaced with medium containing various concentrations of isoliquiritigenin (two-fold serial dilutions from 1000 to 15.63 µM). When virus-infected, untreated cells exhibited approximately 80% cytopathic effect (CPE); 10 µL of CCK-8 solution was added to each well. After incubation at 37 °C for 30 min, absorbance was measured at 450 nm using a microplate reader (Bio-Rad, USA). Viral inhibition rate (%) was calculated using the following formula: (OD _PRV+ ISL_ − OD_PRV_)/(OD_Mock_ − OD_PRV_) × 100%, where OD_PRV+ISL_ represents the OD value of cells treated with isoliquiritigenin and infected with PRV, OD_PRV_ represents the OD value of cells infected with PRV without drug treatment (virus control), and OD_Mock_ represents the OD value of uninfected and untreated cells. The 50% inhibitory concentration (IC_50_) was then calculated by fitting the inhibition rates against drug concentrations using nonlinear regression analysis in GraphPad Prism 9.0 (Dotmatics, Boston, MA, USA). The selectivity index (SI) was calculated as CC_50_/IC_50_.

To evaluate the antiviral activity of isoliquiritigenin at different multiplicities of infection (MOI), PK-15 cells in 96-well plates were infected with PRV at MOIs of 0.01, 0.1, and 1. Following a 1-h adsorption period, the inoculum was removed and replaced with maintenance medium containing 62.5 µM isoliquiritigenin. At 24 h post-infection, total DNA was extracted using DNAiso Reagent (D305; TaKaRa, Dalian, China) according to the manufacturer’s instructions to detect viral DNA copies. For determination of viral gene copies, fluorescent quantitative PCR (FQ-PCR) was performed using a Bio-Rad CFX96 Connect™ Real-Time PCR Detection System (Bio-Rad, Hercules, CA, USA) [24].

### 2.4. Mode of Action Assay

To determine which stage of viral infection was inhibited by isoliquiritigenin, a mode-of-action analysis was conducted using the following five experimental setups [25]. (1) Prevention: The cells were exposed to isoliquiritigenin (62.5 μM) at 37 °C for 1 h, after which they were infected with PRV at a concentration of 100 TCID_50_. (2) Inactivation: The PRV (10,000 TCID_50_) was subjected to an incubation with isoliquiritigenin (62.5 μM) at 37 °C for 1 h. Thereafter, the PRV was diluted to a concentration of 100-fold, and was subsequently introduced to the cells for the purpose of infection. (3) Adsorption: The PRV (100 TCID_50_) was incubated with isoliquiritigenin (62.5 μM) at 4 °C for 1 h prior to infection of the cells. The cells then underwent a thorough washing process to ensure the removal of any unadsorbed virus. Following this, the cells were subjected to an incubation at 37 °C. (4) Penetration: PK-15 cells grown in 6-well plates were pre-chilled at 4 °C for 30 min. The cells were then incubated with PRV (100 TCID_50_) at 4 °C for 1 h to allow viral adsorption. After removal of unadsorbed virus, the cells were gently washed three times with pre-chilled PBS. Pre-chilled maintenance medium containing isoliquiritigenin (62.5 μM) was then added, and the cells were incubated at 37 °C for 1 h to allow viral penetration. Following this incubation, the virus-containing medium was removed, and the cells were washed three times with citrate-citrate sodium buffer (pH 3.0) to inactivate and remove any remaining surface-bound virus. Finally, 2 mL of fresh maintenance medium was added to each well, and the cells were further incubated at 37 °C in a 5% CO_2_ incubator for 48 h. Viral DNA copies were quantified by FQ-PCR as described above. (5) Post-infection: The cells were infected with PRV (100 TCID_50_) at 37 °C for 1 h, then treated with isoliquiritigenin (62.5 μM) after removing unbound virus. After further incubation for 48 h, viral DNA copies were detected by FQ-PCR method described above.

### 2.5. Effect of Isoliquiritigenin on the PRV Growth Curve

PK-15 cells, which had been seeded in 6-well plates, were infected with PRV (MOI = 0.1). After incubation at 37 °C for 1 h, the inoculum was replaced with medium containing isoliquiritigenin (62.5 μM). Cells were harvested at 2, 4, 6, 8, 12, and 18 hpi, and viral DNA was extracted. Viral DNA copies were quantified by fluorescent quantitative PCR (FQ-PCR) as described previously.

### 2.6. Real-Time PCR Assay

Total RNA was extracted from cells cultured in 6-well plates using TRIzol reagent (RA101-01, Biomed, Beijing, China). Reverse transcription was performed using the M-MLV First-Strand cDNA Synthesis Kit (MT403-01, Biomed, Beijing, China). Quantitative real-time PCR was then carried out using the Hieff UNICON Universal Blue qPCR SYBR Master Mix (11184ES08, Yeasen, Shanghai, China) with the primers listed in Table 1. The PCR cycling was performed at 95 °C for 3 min, followed by 40 cycles of 95 °C for 10 s, 59.8 °C for 30 s, and 55 °C for 5 s. The Ct value of the target gene was normalized in relation to the expression of the internal reference gene, β-actin. The relative mRNA expression level of each target gene was calculated according to the 2^−ΔΔCt^ method.

### 2.7. cGAMP Activity Assay

To assess cyclic guanosine monophosphate-adenosine monophosphate (cGAMP) activity, PK-15 cell monolayers infected with PRV (MOI = 1) were treated with 62.5 µM isoliquiritigenin for 1 h. The cells were washed with PBS and then lysed on ice for 30 min. The lysates were centrifuged and the supernatant was collected. Super Nuclease (1000 U/mL; Sino Biological, Beijing, China; Cat. No. SSNP01) was added and incubated at 37 °C for 30 min. The mixture was then heated at 95 °C for 5 min and centrifuged at 12,000 rpm at 4 °C for 5 min. The supernatant was retained and added to PK-15 cells for 6 h. Total RNA was then extracted, and RT-qPCR was performed to measure IFN-β mRNA levels in the recipient cells, serving as an indirect readout of cGAMP activity in the original cell lysates [26].

### 2.8. Western Blotting Assay

PK-15 cells were divided into four groups: Mock, uninfected-untreated group; ISL, uninfected group treated with isoliquiritigenin; PRV, infected-untreated group; PRV + ISL, infected group treated with isoliquiritigenin. Following a 1 h adsorption period, the virus-containing inoculum was removed and replaced with fresh culture medium supplemented with or without 62.5 µM isoliquiritigenin as appropriate. Total proteins were extracted using a commercial kit (BOSTER, Wuhan, China, Cat. No. AR0103-100) at 2, 6, and 12 hpi, respectively. Then, the proteins were separated by 10% sodium dodecyl sulfate-polyacrylamide gel electrophoresis (SDS-PAGE) alongside a pre-stained protein ladder (Chengdu Rongwei Gene Technology Co., Ltd., Cat. No. M201-01, Chengdu, China) with a molecular weight range of 10–180 kDa, and transferred to polyvinylidene difluoride (PVDF) membranes (Millipore, Burlington, MA, USA). The membranes were blocked in 5% (*w*/*v*) skim milk diluted with Tris-buffered saline containing 0.1% (*v*/*v*) Tween 20 (TBST) for 90 min at room temperature, followed by incubation with primary antibodies against IRF3 (1:500, #11312–1-AP; Proteintech, Wuhan, China), p-IRF3 (1:500, #29528–1-AP; Bioss, Beijing, China), STAT1 (1:500, #66545–1-IG; Proteintech, Wuhan, China), p-STAT1 (1:500, #orb7016; Biorobyt, Durham, NC, USA), and β-actin (1:5000, #bs-0061R; Boster, Wuhan, China) at 4 °C overnight. The membranes were washed with TBST and incubated with horseradish peroxidase-conjugated secondary antibody (BA1056; Boster, Wuhan, China, 1:5000) at room temperature for 1.5 h. The proteins were visualized using an enhanced chemiluminescence (ECL) substrate (Bio-Rad, Hercules, CA, USA). The protein expression levels were normalized to β-actin, and the relative band intensities were quantified using ImageJ software (Version 1.47; NIH, Bethesda, MD, USA).

Western blot quantification and normalization: The band intensities were quantified using ImageJ software (Version 1.47; NIH, USA). To correct for variations in total protein loading, the raw integrated density (gray value) values of total IRF3, phosphorylated IRF3 (p-IRF3), STAT1, and p-STAT1 were first normalized to the corresponding β-actin band intensity within the same sample. To assess the activation levels of IRF3 and STAT1, the ratio of phosphorylated to total protein was calculated by dividing the normalized phosphorylated protein value (p-IRF3 or p-STAT1) by the normalized total protein value (IRF3 or STAT1) for each individual sample. The data are presented as the relative protein levels (p-IRF3/IRF3 and p-STAT1/STAT1 ratios). All primary antibodies were purchased from commercial suppliers and have been validated by the manufacturers for cross-reactivity against the relevant species (mouse, pig and monkey) as indicated in the product datasheets. Western blotting was performed, and densitometric quantification was done by investigators blinded to group allocation.

### 2.9. Immunofluorescence Staining in Cultured Cells

PK-15 cells seeded onto glass slides were fixed with 4% paraformaldehyde (PFA) in PBS at room temperature for 15 min. After fixation, the cells were washed three times with PBS for 5 min each. Subsequently, the cells were permeabilized with 0.1% Triton X-100 (Solarbio, Beijing, China) in PBS at room temperature for 10 min. After washing, the cells were blocked with 5% bovine serum albumin (BSA, Beijing Solarbio Science & Technology Co., Ltd., Beijing, China) in PBS at room temperature for 30 min to reduce non-specific binding. The cells were then incubated with anti-p-IRF3 primary antibody (1:200, #29528–1-AP; Bioss, Beijing, China) overnight at 4 °C. After washing with PBS, the cells were incubated with CoraLite594-conjugated secondary antibody (SA00013–4; Proteintech, Wuhan, China, 1:100) at room temperature for 1 h in the dark. After washing, the nuclei were counterstained with DAPI (1 μg/mL in PBS; Beijing Solarbio Science & Technology Co., Ltd., Beijing, China) for 5 min, followed by a final wash with PBS. The slides were mounted with an anti-fade mounting medium and coverslipped. Images were acquired using a fluorescence microscope (Olympus VS200, Evident Scientific, Shanghai, China). IF staining analysis was performed and quantified by operators blinded to the experimental groups. Relative fluorescence intensities were quantified using Fiji (an enhanced distribution of ImageJ, Version 1.47; NIH, Bethesda, MD, USA).

### 2.10. STAT1 Gene Silencing Method

STAT1 siRNA was designed and synthesized by Youkang Biotechnology Co., Ltd. (Chengdu, China). The targeting sequence for transient silencing was 5′-GCACGGUGAUGUUAGACAATT-3′ (sense) and 3′-UUGUCUAACAUCACCGUGCTT-5′ (antisense), which was specifically designed and validated for targeting Vero cell STAT1 mRNA. The non-targeting siRNA sequence was 5′-UUCUCCGAACGUGUCACGUTT-3′ (sense) and 5′-ACGUGACACGUUCGGAGAATT-3′ (antisense). Both siRNAs were specifically designed and validated by Youkang Biotechnology Co., Ltd., Chengdu, China. (The non-targeting siRNA served as the negative control.) In addition, a transfection reagent-only control group (cells treated with Lipofectamine 2000 alone, without any siRNA) was included. The siRNAs were transfected into subconfluent (approximately 50%) Vero cell monolayers using Lipofectamine 2000 (Cat. No. 11668030; Thermo Fisher Scientific, Waltham, MA, USA). Cells were incubated at 37 °C with 5% CO_2_ for 24 h. The transfection medium was then replaced with maintenance medium containing PRV (MOI = 1), with or without isoliquiritigenin. Following a 12 h incubation, the total cellular protein was extracted in accordance with the manufacturer’s protocol (Wuhan Boster, AR0103-100, Wuhan, China). Subsequently, a Western blot analysis was conducted as described above.

### 2.11. Animals, Experimental Design and Sample Collection

Forty specific pathogen-free (SPF) male KM mice weighing 20 ± 2 g were purchased from Chengdu Dossy Experimental Animals Co., Ltd. The mice were acclimated to the experimental environment for 7 days prior to the commencement of the study. Mice were housed in an SPF facility, 5 mice per cage, under a 12 h light/12 h dark cycle at 22 ± 2 °C and 50 ± 10% humidity, with food and water ad libitum. All procedures involving animals and their care in this study were approved by the Ethics Committee of Sichuan Agricultural University (Approval No. 20251109) and were conducted in strict accordance with the Regulations on the Management of Laboratory Animals (State Science and Technology Commission of China, No. 2, 1988) and the Interim Measures for the Management of Laboratory Animals in Sichuan Province (Sichuan Provincial Department of Science and Technology, China, No. 25, 2013). Mice were randomly divided into four groups (*n* = 10): an uninfected-untreated group (Mock), an uninfected group treated with isoliquiritigenin (ISL), an infected-untreated group (PRV), and an infected group treated with isoliquiritigenin (PRV + ISL). The Mock group received the vehicle (0.5% CMC-Na) by oral gavage at the same volume and according to the same schedule (once daily for five consecutive days) as the ISL-treated groups. The dose of 50 mg/kg was selected based on preliminary dose-finding experiments, in which 50, 100, and 200 mg/kg were tested; the 50 mg/kg dose exhibited the most favorable balance between antiviral efficacy and safety in mice and was therefore chosen for all subsequent in vivo experiments. All treatments were administered via intragastric gavage. The mice in the isoliquiritigenin-treated groups received isoliquiritigenin via oral gavage for five consecutive days. The treatment protocol consisted of two phases: a preventive phase and a therapeutic phase. During the preventive phase (days 1–5), mice in the ISL and PRV+ISL groups received 50 mg/kg isoliquiritigenin once daily by oral gavage for five consecutive days, while those in the Mock and PRV groups received an equal volume of vehicle (0.5% CMC-Na). On day 5, mice in the PRV and PRV+ISL groups were intraperitoneally challenged with 0.1 mL of PRV at 2 × 10^4^ TCID_50_, following the protocol established in our previous study [27]. At this dose, the mice exhibited stable infection; the Mock and ISL groups received an equal volume of vehicle via intraperitoneal injection. After viral challenge, the therapeutic phase was initiated: the ISL and PRV+ISL groups continued to receive the same dose of isoliquiritigenin (50 mg/kg) daily by gavage for an additional 4 days (days 6–9), whereas the Mock and PRV groups received an equal volume of 0.5% CMC-Na, until the endpoint of the experiment. Humane endpoints were strictly defined for early euthanasia of moribund animals. Mice were monitored at least twice daily for signs of morbidity, including severe lethargy, hunched posture, labored breathing, paralysis, and inability to reach food or water. Animals meeting any of these criteria were humanely euthanized by cervical dislocation prior to the endpoint to minimize suffering. Mortality was recorded daily by investigators blinded to the group allocation. At 4 days post-infection (dpi), the mortality rate in the PRV group reached 70%, which exceeded the predefined humane endpoint threshold (≥50%) and prompted early termination of the experiment. All surviving mice in each group were then euthanized by cervical dislocation at this unified endpoint (4 dpi) to ensure consistency in sample collection timing. Tissues from the brain, kidneys, heart, liver, lungs, and spleen were collected, and then subjected to cryogenic homogenization in liquid nitrogen, and total RNA was extracted using the TRIzol method as described above. The total proteins of brain and kidney specimens (approximately 100 mg) were processed according to the protocol stipulated by the manufacturer (Wuhan Boster, AR0101-30, Wuhan, China) for the purpose of Western blotting analysis, which was performed and quantified by operators blinded to the experimental groups.

### 2.12. Immunofluorescence Staining of Tissue Sections

Brain and kidney specimens were fixed in 4% paraformaldehyde for 24 h. Targeted sections of the brain and kidneys were then excised, dehydrated, embedded in paraffin, and sectioned. After deparaffinization and rehydration, the sections were treated with 0.2% Triton X-100. Antigen retrieval was performed by heating the sections in sodium citrate buffer using a microwave. The sections were then blocked with 3% BSA for 40 min. For immunofluorescence, sections were incubated with anti-p-IRF3 primary antibody (1:200, #29528-1-AP; Bioss, Beijing, China) overnight at 4 °C, followed by incubation with CoraLite594-conjugated secondary antibody (SA00013-4; Proteintech, Wuhan, China; 1:100) for 1 h at room temperature in the dark. Nuclei were counterstained with DAPI. Images were acquired using a fluorescence microscope. IF staining analysis was performed and quantified by operators blinded to the experimental groups.

### 2.13. Statistical Analysis

All data are expressed as mean ± SD from at least three independent biological replicates. Statistical analyses were performed using GraphPad Prism 9.0. One-way or two-way analysis of variance (ANOVA) was applied as appropriate, followed by Tukey’s post hoc test for multiple comparisons. Differences were considered statistically significant at *p* < 0.05, *p* < 0.01, and *p* < 0.001. For the animal survival experiment, the Kaplan–Meier method with the log-rank (Mantel–Cox) test was used to compare survival curves between groups.

## 3. Results

### 3.1. Cytotoxicity and Anti-PRV Activity of Isoliquiritigenin

**Figure 1:**
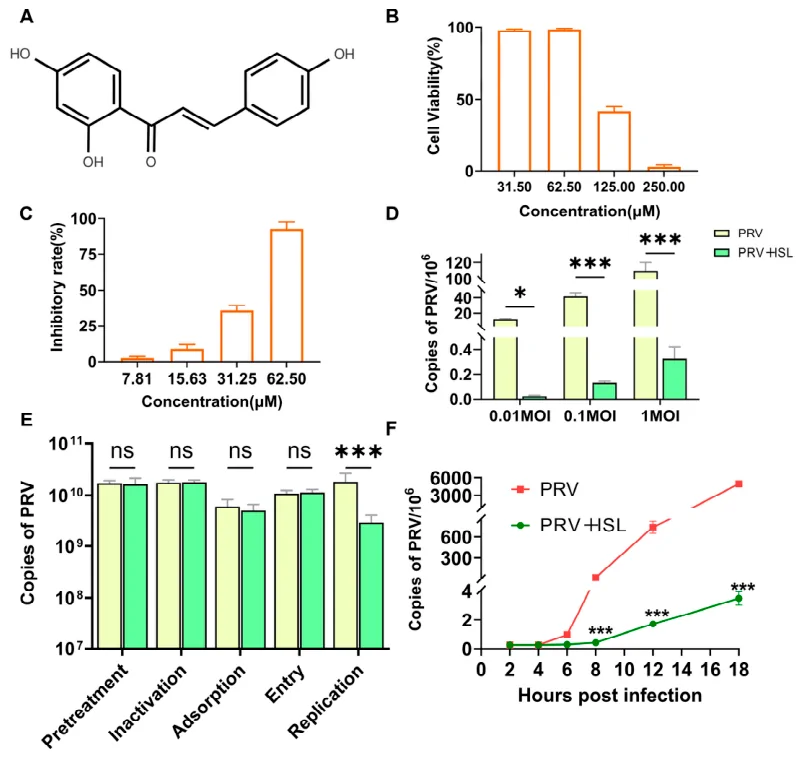
Anti-PRV Activity of isoliquiritigenin. (**A**) Chemical structure of isoliquiritigenin. (**B**) Cytotoxicity of isoliquiritigenin in PK-15 cells. (**C**) Inhibitory rate of PRV by isoliquiritigenin. (**D**) Antiviral activity at different MOIs. (**E**) Mode of action analysis. (**F**) Growth curve of PRV in PK-15 cells. PRV, infected-untreated group; PRV + ISL, infected group treated with isoliquiritigenin. *, *** indicate *p* < 0.05, and *p* < 0.001, respectively, when compared with the infected-untreated group. ns, not significant. In Figure 1 (**D**,**F**), “Copies of PRV/10^6^” denotes the unit of measurement, and the Y-axis break (indicated by//) is used to facilitate visualization of the large differences in viral copy numbers across groups.

Throughout all experiments, the final concentration of DMSO in the culture medium was maintained below 0.5%, a level that has no measurable effect on cell viability or viral replication. Cytotoxicity assessment demonstrated that treatment with 125 μM isoliquiritigenin resulted in an approximately 50% reduction in cell viability (Figure 1B). No significant cytotoxic effects were observed on PK-15 cells at concentrations up to 62.5 μM, which was therefore established as the maximum non-cytotoxic concentration for subsequent antiviral experiments. All subsequent antiviral and mechanistic assays were performed at this concentration. Isoliquiritigenin exhibited a dose-dependent inhibitory effect against PRV in the IC_50_ assay (Figure 1C). At 62.5 μM, isoliquiritigenin almost completely abolished PRV-induced cytopathic effects (Figure 1C). The half-maximal inhibitory concentration (IC_50_) of isoliquiritigenin against PRV was determined to be 35.47 μM, and the half-maximal cytotoxic concentration (CC_50_) was 118.3 μM, yielding a selective index (SI = CC_50_/IC_50_) of 3.34. To further assess the robustness of its antiviral activity, the initial infection dose was increased to 0.01, 0.1, and 1 MOI. Under these conditions, isoliquiritigenin at 62.5 μM still exhibited significant antiviral effects, with viral gene copy numbers being significantly reduced even at an MOI of 1 (Figure 1D).

### 3.2. Mode of Action

To elucidate the antiviral mode of action, a series of experiments were performed targeting distinct stages of the viral replication cycle. To facilitate visualization, viral copy numbers in Figure 1E are presented as Log_10_-transformed values. As shown in Figure 1E, isoliquiritigenin at 62.5 μM exerted a significant inhibitory effect specifically during the viral replication stage, whereas no protective effect on cells was observed during other phases of the viral cycle. Analysis of viral growth curves revealed that isoliquiritigenin did not significantly affect viral replication within the first 8 hpi; however, marked suppression of viral growth was observed between 8 and 18 hpi, with viral gene copy numbers being significantly reduced (Figure 1F). Collectively, these results indicate that the antiviral effect of isoliquiritigenin was primarily attributable to its ability to inhibit viral replication.

### 3.3. The Regulatory Role of Isoliquiritigenin on the Type I Interferon Signaling Pathway Following PRV Infection

**Figure 2:**
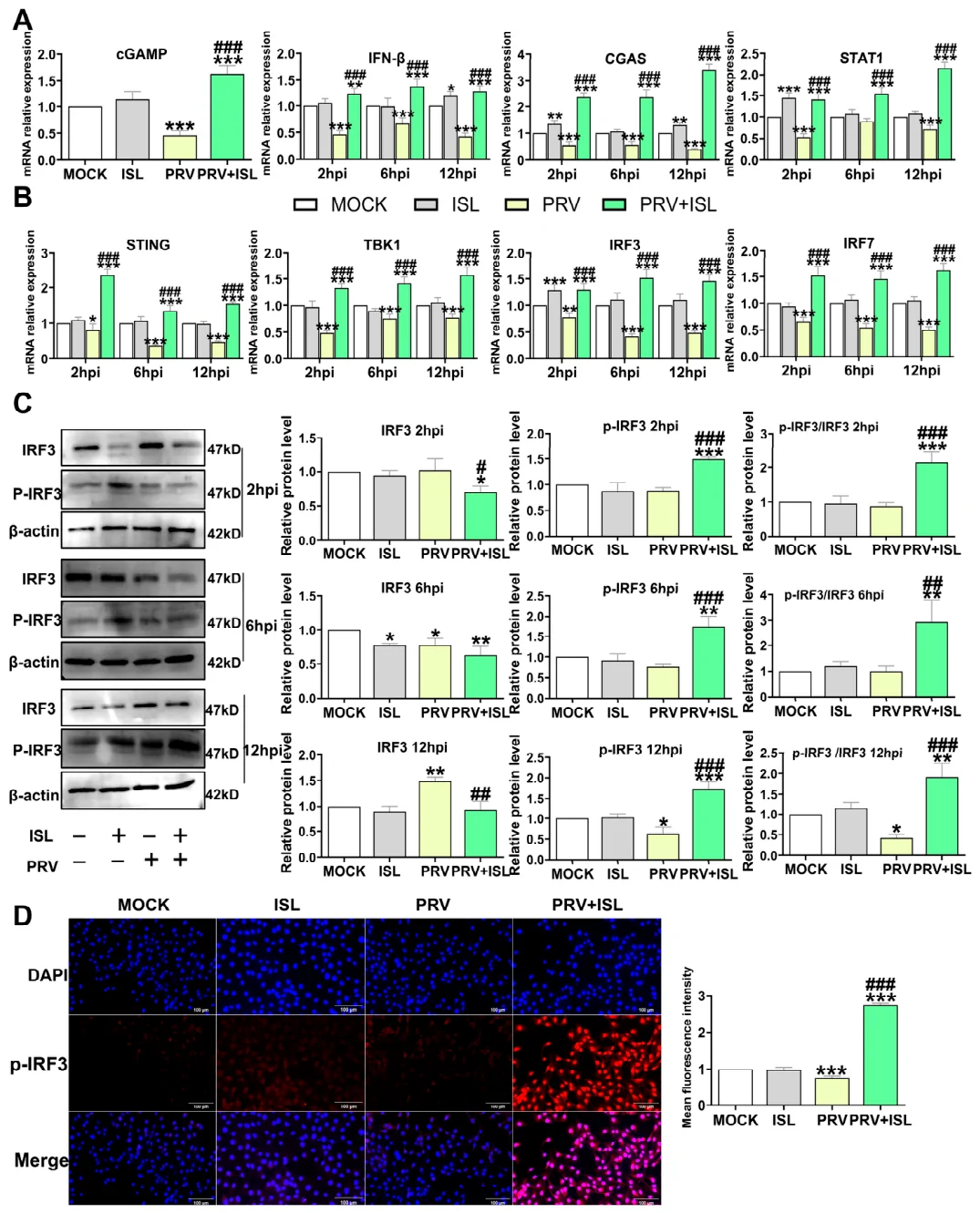
Regulation of the cGAS/STING pathway by isoliquiritigenin in PRV-infected PK-15 cells. (**A**) Effects of isoliquiritigenin on cGAMP activity and type I interferon pathway gene expression. To assess the functional activity of cGAMP in response to isoliquiritigenin treatment, cell extracts from cells with or without isoliquiritigenin treatment were transferred to untreated recipient cells, and the mRNA levels of IFN-β were measured (*n* = 6). (**B**) Effects of isoliquiritigenin on transcriptional levels of STING, TBK1, IRF3, and IRF7 at 2, 6, and 12 hpi (*n* = 6). (**C**) Protein expressions of IRF3 and p-IRF3 at 2, 6, and 12 hpi (*n* = 3). (**D**) Immunofluorescence detection of p-IRF3 protein expression in PK15 cells (*n* = 3). Mock, uninfected-untreated group; ISL, uninfected group treated with isoliquiritigenin; PRV, infected-untreated group; PRV + ISL, infected group treated with isoliquiritigenin. All qPCR data were normalized to β-actin as the internal reference gene and presented as relative expression levels, with the Mock group set to 1. All data are expressed as mean ± SD. *, **, *** indicate *p* < 0.05, *p* < 0.01, and *p* < 0.001, respectively, when compared with the mock group. #, ##, and ### indicate *p* < 0.05, *p* < 0.01, and *p* < 0.001, respectively, when compared with the PRV group.

The cGAS/STING pathway serves as a critical upstream signaling cascade responsible for initiating type I interferon production. Upon activation of this pathway, the second messenger cGAMP is synthesized. To assess cGAMP activity, cellular extracts were prepared and transferred to naïve recipient cells, and IFN-β gene expression was measured as an indirect readout of cGAMP activity [28]. As shown in Figure 2A, the cGAMP-stimulated IFN-β response was significantly reduced in the PRV group compared to the Mock group. In contrast, treatment with isoliquiritigenin markedly enhanced this IFN-β response, indicating increased cGAMP activity relative to the Mock group. These findings suggest that isoliquiritigenin may potentiate cGAS/STING signaling activation following PRV infection.

Next, the regulatory effect of isoliquiritigenin on the expression of key components of the type I interferon signaling pathway was investigated. As shown in Figure 2A, PRV infection significantly downregulated the mRNA levels of cGAS, STAT1, and IFN-β compared with the Mock group. However, treatment with isoliquiritigenin markedly restored the expression of these genes, with levels surpassing those observed in the Mock group. These findings indicate that isoliquiritigenin effectively reactivates the type I interferon signaling pathway following PRV infection.

### 3.4. The Regulatory Effect of Isoliquiritigenin on the cGAS/STING Signaling Pathway

The transcriptional levels of key components of the cGAS/STING signaling pathway, including STING, TBK1, IRF3, and IRF7, were assessed at 2, 6, and 12 hpi (Figure 2B). Compared to Mock group, PRV infection significantly downregulated the mRNA expression of these genes. However, treatment with isoliquiritigenin was associated with increased transcript levels, which not only exceeded those of the PRV group but also surpassed the baseline levels observed in Mock group cells. Upon activation of the cGAS/STING pathway, IRF3 undergoes phosphorylation and dimerization, subsequently translocating to the nucleus to initiate IFN-β transcription and the expression of downstream interferon-stimulated genes. Accordingly, the protein levels of total IRF3 and p-IRF3 were examined at 2, 6, and 12 hpi (Figure 2C). The p-IRF3/IRF3 ratio in PRV-infected cells showed no significant alteration during the early stages of infection but declined markedly by 12 hpi. In contrast, isoliquiritigenin treatment was associated with significantly elevated p-IRF3 levels and p-IRF3/IRF3 ratios at all three time points examined. These findings suggest that PRV infection leads to suppression of the cGAS/STING pathway at 12 hpi, whereas isoliquiritigenin correlates with sustained activation of this pathway throughout the course of infection. Indirect immunofluorescence analysis of p-IRF3 further corroborated these observations, demonstrating a marked increase in IRF3 phosphorylation upon isoliquiritigenin treatment compared to the PRV group (Figure 2D). Collectively, these results suggest that while PRV infection suppresses the cGAS/STING signaling pathway as viral replication progresses, isoliquiritigenin treatment is associated with maintained activation of this pathway during PRV infection.

### 3.5. The Regulatory Role of Isoliquiritigenin in the JAK/STAT Signaling Pathway Following PRV Infection

**Figure 3:**
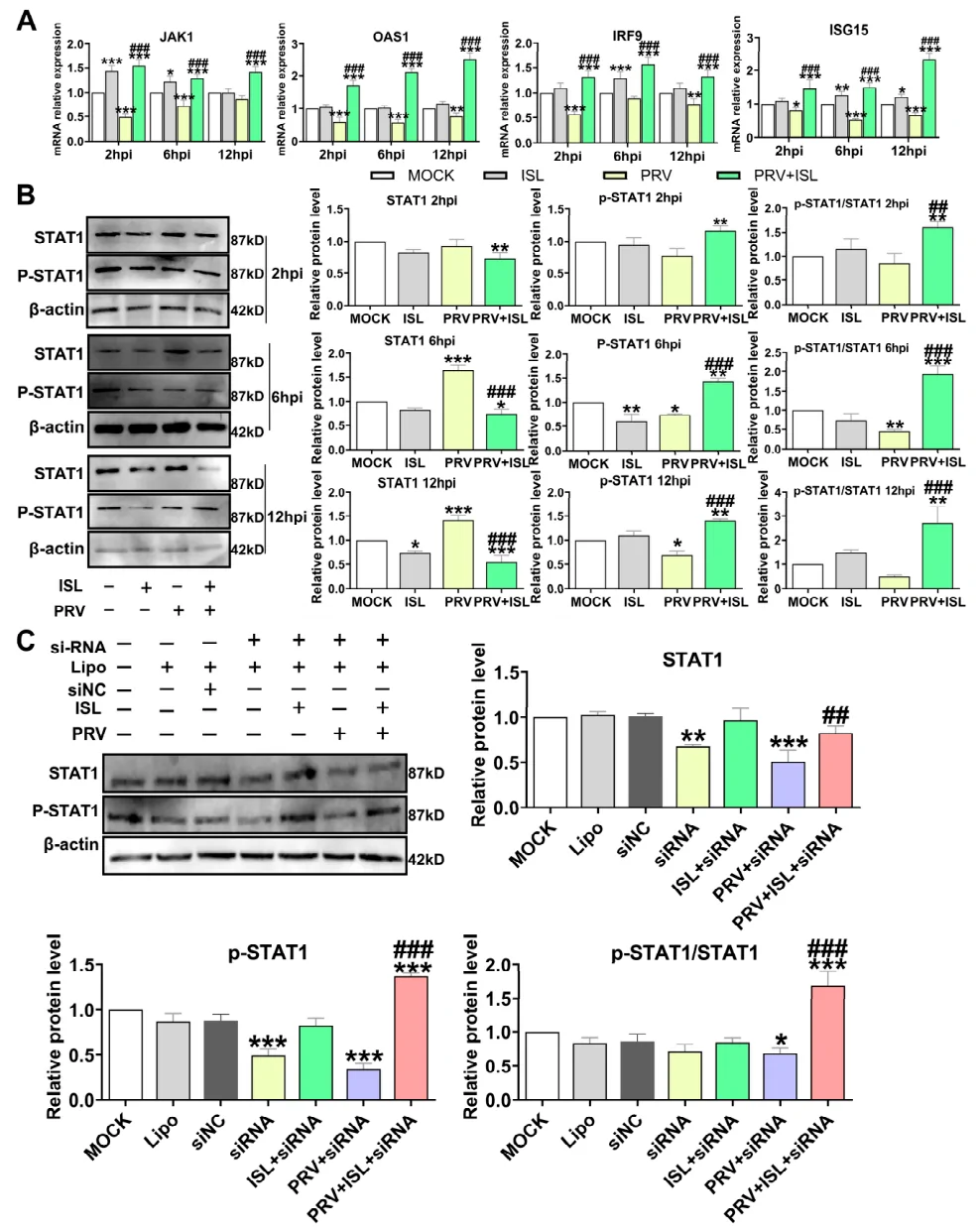
Regulatory role of isoliquiritigenin in the JAK/STAT signaling pathway following PRV infection in PK-15 and Vero cells. (**A**) Effects of isoliquiritigenin on transcriptional levels of JAK1, OAS1, IRF9, and ISG15 at 2, 6, and 12 hpi in PK15 cells (*n* = 6). (**B**) Protein expression of STAT1 and p-STAT1 proteins at 2, 6, and 12 hpi in PK15 cells (*n* = 3). (**C**) Protein expression of STAT1 and p-STAT1 proteins in Vero cells (*n* = 3). All data are expressed as mean ± SD. Mock, uninfected-untreated group; ISL, uninfected group treated with isoliquiritigenin; PRV, infected-untreated group; PRV + ISL, infected group treated with isoliquiritigenin; Lipo, group with transfection reagent only; siNC, uninfected group transfected with non-targeting siRNA; siRNA, uninfected with siRNA; ISL + siRNA, uninfected group treated with siRNA and isoliquiritigenin; PRV + siRNA, infected-untreated group with siRNA; PRV + ISL + siRNA, infected group treated with isoliquiritigenin and siRNA. All qPCR data were normalized to β-actin as the internal reference gene and presented as relative expression levels, with the Mock group set to 1. *, **, *** indicate *p* < 0.05, *p* < 0.01, and *p* < 0.001, respectively, when compared with the mock group. ## and ### indicate *p* < 0.01, and *p* < 0.001, respectively, when compared with the PRV group (A, B) or the PRV + siRNA group (C).

Upon activation of the cGAS/STING pathway, interferons are secreted into the extracellular space and bind to their cognate receptors, IFNAR1 and IFNAR2, on the cell membrane, thereby triggering the JAK/STAT signaling cascade. This cascade ultimately drives the expression of antiviral effector proteins known as ISGs, which mediate the observed antiviral effects [29]. To assess the impact of PRV infection and isoliquiritigenin treatment on this pathway, the transcriptional levels of key JAK/STAT pathway components, including JAK1, OAS1, IRF9, and ISG15, were quantified. As shown in Figure 3A, PRV infection significantly suppressed the mRNA expression of these genes compared to uninfected controls. Given that STAT1 and its phosphorylation status are pivotal to JAK-STAT signaling, the protein levels of total STAT1 and p-STAT1 were examined at 2, 6, and 12 hpi (Figure 3B). The p-STAT1/STAT1 ratio in PRV-infected cells showed a decrease. In contrast, isoliquiritigenin treatment significantly elevated both p-STAT1 levels and the p-STAT1/STAT1 ratio at all three time points examined, with values consistently exceeding those of the other three groups. These findings suggest that the JAK/STAT signaling pathway is suppressed during PRV infection. Notably, isoliquiritigenin treatment was associated with sustained activation of this pathway throughout the course of infection, as evidenced by persistently elevated p-STAT1/STAT1 ratios. Collectively, these results suggest that isoliquiritigenin is associated with maintained activation of the JAK/STAT signaling pathway during PRV infection.

### 3.6. Validation of Isoliquiritigenin in Activating the Type I Interferon Signaling Pathway Following PRV Infection in Vero Cells

To investigate the role of STAT1 in the antiviral activity of isoliquiritigenin, STAT1 knockdown assays were performed in Vero cells. Vero cells were chosen for their natural deficiency in type I interferon production, allowing us to assess the direct role of STAT1 without confounding effects from upstream interferon signaling. As a downstream effector of the type I interferon pathway, the expression level and phosphorylation status of STAT1 serve as reliable indicators of pathway activation. The following control groups were included: Lipo (group with transfection reagent only) and siNC (uninfected group transfected with non-targeting siRNA). None of these control groups showed significant differences in STAT1 expression or p-STAT1/STAT1 ratio compared with the Mock group (uninfected-untreated control), confirming that the observed effects were specifically attributable to STAT1 knockdown rather than to transfection or non-specific siRNA effects. In cells transfected with STAT1 siRNA prior to PRV infection, STAT1 protein expression was significantly reduced compared with the blank group, accompanied by a decrease in the p-STAT1/STAT1 ratio. (Figure 3C). Similarly, in cells transfected with STAT1 siRNA following PRV infection, STAT1 protein expression was more markedly downregulated, accompanied by a significantly decrease in the p-STAT1/STAT1 ratio. These results suggest that STAT1 siRNA was associated with suppression of JAK/STAT pathway activation in PRV-infected cells. Notably, following isoliquiritigenin treatment under siRNA transfection conditions, the p-STAT1/STAT1 ratio was markedly elevated relative to the blank group, suggesting that isoliquiritigenin may be associated with restoration of the previously inhibited JAK/STAT pathway (Figure 3C).

### 3.7. Isoliquiritigenin Activated the Type I Interferon Signaling Pathway in Mice Infected with PRV

**Figure 4:**
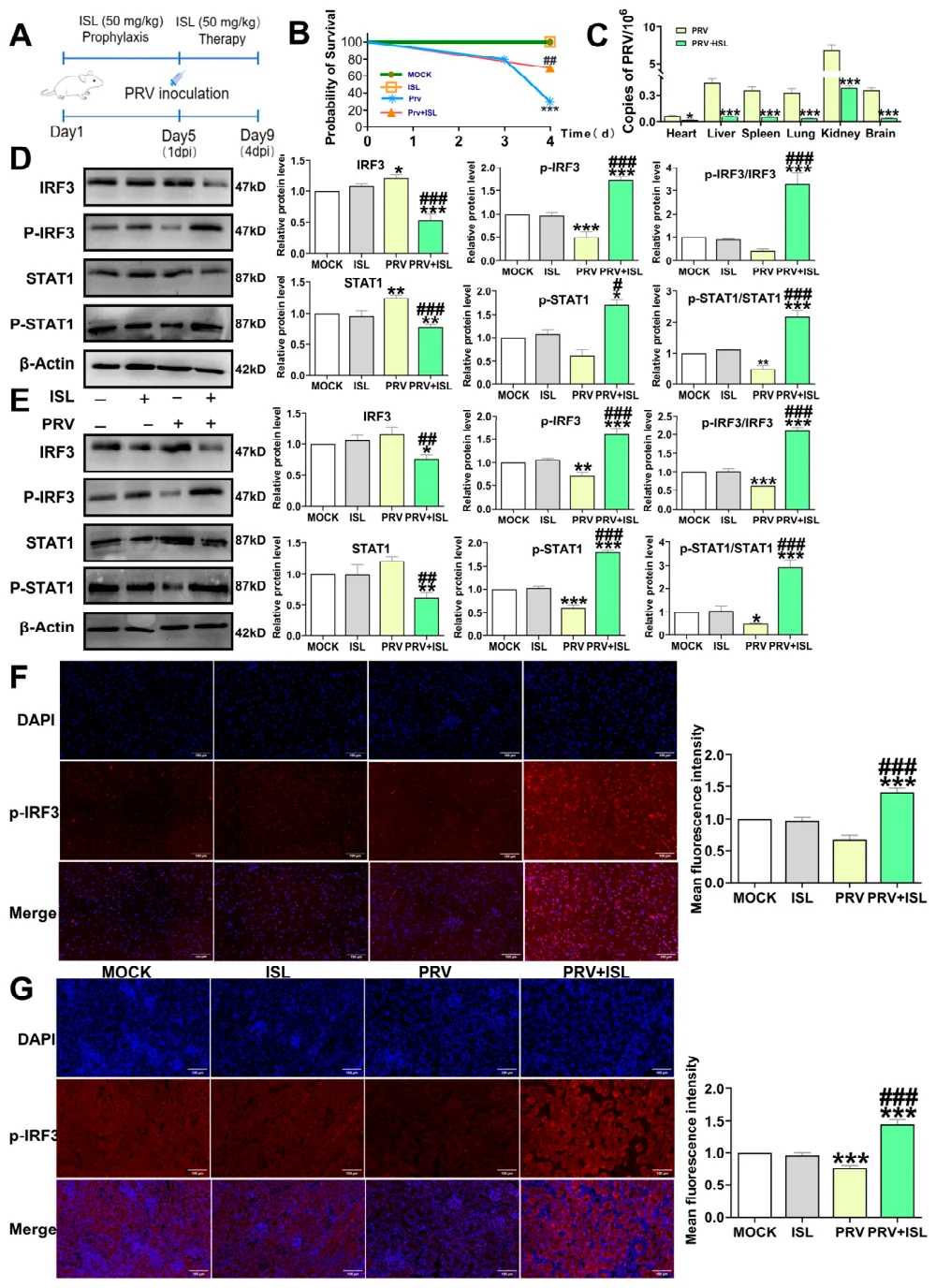
Antiviral activity of isoliquiritigenin in PRV-infected mice and its effects on the cGAS/STING and JAK/STAT signaling pathways in vivo. (**A**) Schematic diagram of the experimental animal dosing and challenge protocol. (**B**) Survival rates of mice in each group depicted by Kaplan–Meier survival curves (*n* = 10). (**C**) Virus load of heart, liver, spleen, lung, kidney, and brain in each group (*n* = 3). (**D**) Protein expression of the cGAS/STING and JAK/STAT signaling pathways in the brain of mice (*n* = 3). (**E**) Protein expression of the cGAS/STING and JAK/STAT signaling pathways in the kidney of mice (*n* = 3). (**F**) Immunofluorescence detection of p-IRF3 expression in the brain of mice (*n* = 3). (**G**) Immunofluorescence detection of p-IRF3 expression in the kidney of mice (*n* = 3). All data are expressed as mean ± SD. Mock, uninfected-untreated group; ISL, uninfected group treated with isoliquiritigenin; PRV, infected-untreated group; PRV + ISL, infected group treated with isoliquiritigenin. *, **, *** indicate *p* < 0.05, *p* < 0.01, and *p* < 0.001, respectively, when compared with the mock group. #, ##, and ### indicate *p* < 0.05, *p* < 0.01, and *p* < 0.001, respectively, when compared with the PRV group.

**Figure 5:**
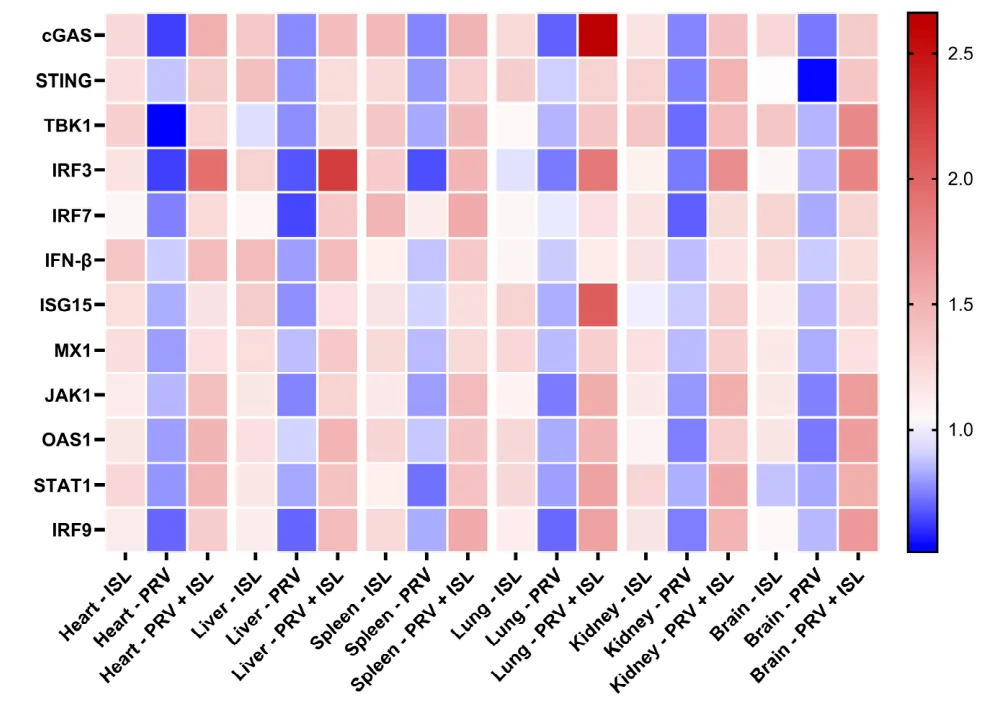
Heatmap analysis of ISL-regulated gene expression across different organs. Mock, uninfected-untreated group; ISL, uninfected group treated with isoliquiritigenin; PRV, infected-untreated group; PRV + ISL, infected group treated with isoliquiritigenin. The heatmap displays the relative mRNA expression levels of 12 genes (rows) across six different organs (columns): Heart, Liver, Spleen, Lung, Kidney, and Brain. Within each organ, the columns represent the four treatment groups as indicated in the column labels. The color scale represents the fold change relative to the Mock control (Mock = 1.0, set as white), with red indicating upregulation and blue indicating downregulation; color intensity increases progressively with the magnitude of the fold change. All data are presented as the means of six biological replicates per group (*n* = 6).

**Figure A1:**
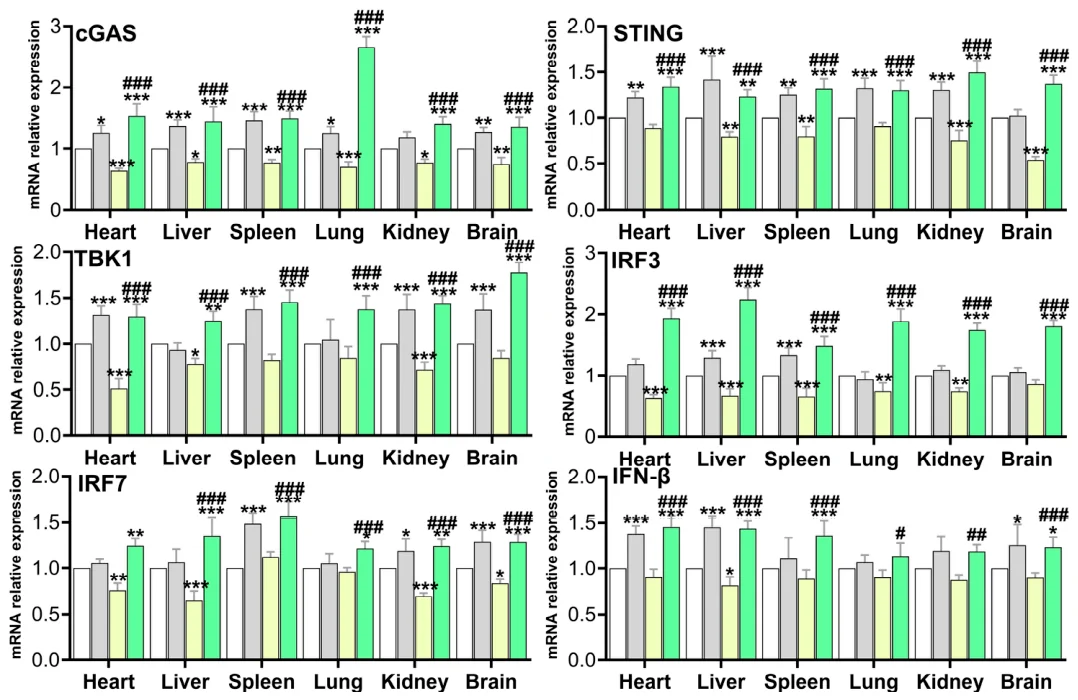

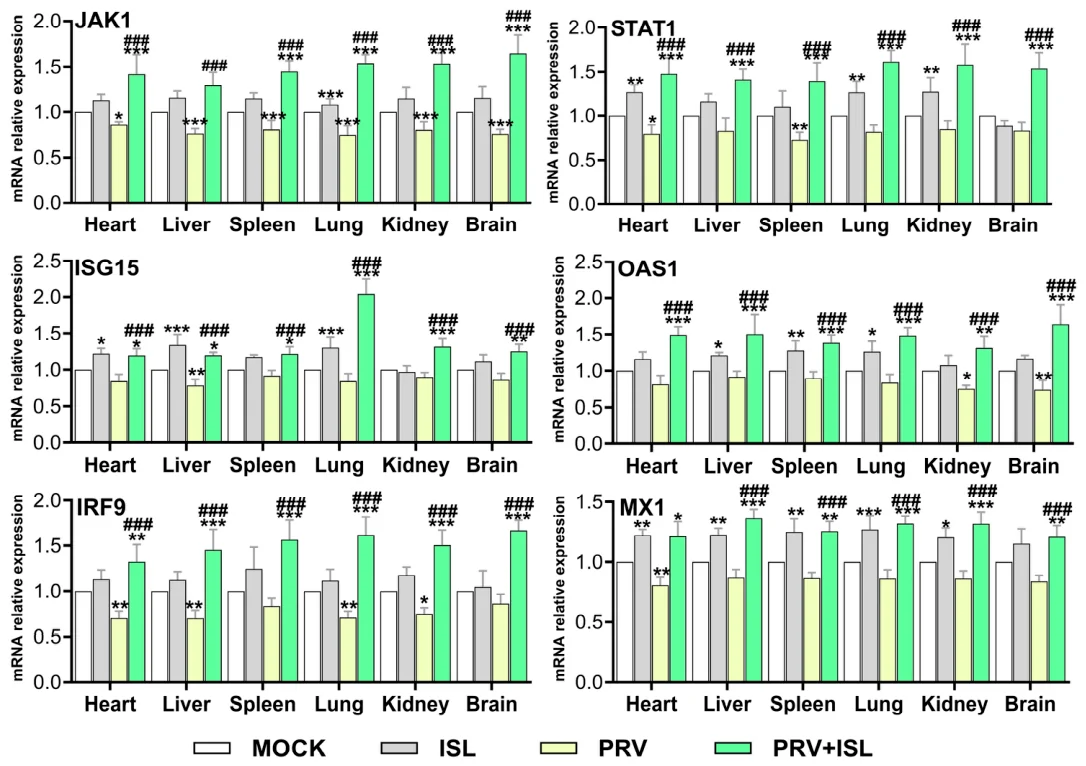
Effects of isoliquiritigenin on the transcriptional levels of cGAS, STING, TBK1, IRF3, IRF7, IFN-β, JAK1, STAT1, ISG15, OAS1, IRF9, and MX1 via the cGAS/STING and JAK/STAT signaling 8pathways in PRV-infected mice (*n* = 6). All qPCR data were normalized to β-actin as the internal reference gene and presented as relative expression levels, with the Mock group set to 1. Mock, uninfected-untreated group; ISL, uninfected group treated with isoliquiritigenin; PRV, infected-un- treated group; PRV + ISL, infected group treated with isoliquiritigenin. *, **, *** indicate *p* < 0.05, *p* < 0.01, and *p* < 0.001, respectively, when compared with the mock group. #, ##, and ### indicate *p* < 0.05, *p* < 0.01, and *p* < 0.001, respectively, when compared with the PRV group.

To further evaluate the in vivo antiviral efficacy of isoliquiritigenin against PRV, its protective effects were assessed in a PRV-infected mouse model. PRV-infected mice exhibited prominent clinical signs, including wet corners of the mouth with salivation, purulent eye lesions with adhesion, scratching at the injection site, lethargy, labored breathing, and a tendency to huddle near the water dispenser. These symptoms were first observed at 3 dpi. As shown in Figure 4B, mortality in the PRV group began at 3 dpi, with a 10% mortality rate that increased to 70% by 4 dpi. In contrast, isoliquiritigenin-treated mice developed only mild purulent eye lesions at 3 dpi, with deaths first occurring at 4 dpi and a final mortality rate of 30%, corresponding to a 40-percentage-point reduction compared to the PRV-only group (70% to 30%). These findings suggest that isoliquiritigenin increases the survival rate in PRV-infected mice. Viral replication in various tissues was subsequently quantified by FQ-PCR. As illustrated in Figure 4C, PRV copy numbers were highest in the kidneys, followed by the liver, brain, spleen, and lungs, with the lowest levels detected in the heart. Notably, isoliquiritigenin treatment significantly reduced viral loads across all examined organs, with the most pronounced decrease in viral gene copy numbers observed in the kidneys, demonstrating its potent inhibitory effect on PRV replication in vivo. Given that the brain and kidneys are established primary target organs for PRV, the protein expression levels of IRF3, p-IRF3, STAT1, and p-STAT1 in these tissues were further examined. Following PRV infection, the p-IRF3/IRF3 and p-STAT1/STAT1 ratios in the brain and kidneys were reduced. However, isoliquiritigenin treatment markedly elevated these ratios, suggesting enhanced activation of the type I interferon pathway (Figure 4D,E). These observations were corroborated by immunofluorescence analysis of brain and kidney tissues (Figure 4F,G). To gain further mechanistic insight, the transcriptional levels of key components of the type I interferon signaling pathway, including cGAS, STING, TBK1, IRF3, IRF7, IFN-β, JAK1, STAT1, ISG15, OAS1, IRF9, and MX1, were analyzed across multiple tissues (heart, liver, spleen, lung, kidney, and brain) (Figure 5). Compared to uninfected controls, PRV-infected mice exhibited a general downregulation of these genes. In contrast, isoliquiritigenin treatment significantly upregulated their expression, with levels exceeding both Mock and PRV groups. Detailed statistical significance analysis is provided in Figure A1. Collectively, these results suggest that PRV evades the host immune response by suppressing type I interferon signaling, whereas isoliquiritigenin is associated with activation of the pathway.

## 4. Discussion

In the present study, we report that isoliquiritigenin exhibits antiviral activity against PRV both in vitro and in vivo. Mechanistically, we found that PRV infection significantly reduced intracellular cGAMP levels and suppressed the transcriptional expression of key genes including STING, TBK1, IRF3, and IRF7, while isoliquiritigenin treatment effectively reversed these effects and restored cGAMP content. Additionally, isoliquiritigenin treatment enhanced IRF3 and STAT1 phosphorylation and upregulated the expression of multiple ISGs. These findings suggest that isoliquiritigenin may exert its antiviral effects against PRV in a manner associated with modulation of the cGAS/STING and JAK/STAT signaling cascades. Beyond its direct impact on these innate immune cascades, isoliquiritigenin has been reported to exert cytoprotective and immunomodulatory effects via activation of the Nrf2-dependent antioxidant pathway and suppression of the NF-κB-mediated inflammatory response, thereby mitigating BDE-47(2,2′,4,4′-tetrabromodiphenyl ether)-induced oxidative stress, apoptosis, and immune dysfunction [30]. This suggests that the anti-PRV activity of isoliquiritigenin may arise from the synergistic coordination of multiple signaling pathways, which, together with its intrinsic anti-inflammatory and antioxidant properties, constitutes the molecular basis for its potent antiviral efficacy both in vitro and in vivo.

The antiviral activity of a compound is commonly evaluated by two key parameters: the IC_50_ and SI. The IC_50_ of isoliquiritigenin against PRV was determined to be 35.47 μM, with a SI of 3.34. By comparison, a flavonoid mixture derived from *Ocotea notata* leaves was reported to exhibit SI values of 5.5 against HSV-1 and 8.5 against HSV-2 [31], whereas quercetin demonstrated an SI exceeding 20 against HSV-1 [32]. In this context, the SI of isoliquiritigenin is relatively modest, suggesting a moderate selectivity and a somewhat narrow therapeutic window. In antiviral drug discovery, an SI below 10 generally indicates a narrow therapeutic window, meaning that the effective concentration is close to the cytotoxic concentration. Importantly, the SI is highly dependent on cell type, virus strain, and assay conditions; direct comparisons across studies must be made with caution. In addition, a moderate SI does not preclude further development—natural product leads with SI values in the single digits have been successfully optimized through medicinal chemistry, formulation, or combination strategies. This principle is exemplified by the optimization of betulinic acid, a natural pentacyclic triterpenoid that initially exhibited limited potency and weak selectivity. Through systematic amidation modifications, researchers successfully transformed betulinic acid into a lead candidate with improved selective toxicity (compound 9; HeLa: IC_50_ = 5.4 μM, SI > 9.3; MCF-7: IC_50_ = 7.0 μM, SI > 7.2). This case provides a compelling precedent for the notion that natural product leads with modest SI values can be advanced through rational structural optimization, supporting the feasibility of similar strategies for isoliquiritigenin [33]. Notably, the compound demonstrated significant in vivo efficacy in our mouse model (a 40-percentage-point reduction in mortality), suggesting that despite the narrow in vitro window, sufficient exposure can be achieved to exert antiviral effects. Since no PRV-specific drug is currently available on the market, a direct head-to-head comparison with an approved antiviral agent is not feasible. However, isoliquiritigenin can be compared with other natural products that possess anti-herpesvirus activity. For instance, resveratrol has been reported to inhibit PRV with an IC_50_ of 17.17 μM (SI > 15.31) [], and kaempferol with an IC_50_ of 25.57 μM (SI 9.97) [25], both exhibiting anti-PRV activity in PK-15 cells. In comparison, isoliquiritigenin (IC_50_ = 62.5 µM, SI 3.34) shows relatively weaker antiviral activity. This may be due to differences in chemical structure, which could affect its binding affinity to target proteins. Nonetheless, this does not diminish its value as a promising lead compound for anti-PRV drug development. Rather than positioning it as an immediate clinical candidate, isoliquiritigenin should be considered a viable starting point for structural optimization to improve its potency and selectivity. Future efforts in rational derivatization, formulation development, or combination therapy with other antiviral agents are expected to further enhance its therapeutic utility against PRV infection.

The importance of the cGAS/STING pathway in antiviral defense is well documented. Kombu polysaccharide enhances cGAS/STING signaling during HSV-1 infection by stabilizing STING protein through inhibition of autophagic degradation [34], and cGAS knockout mice exhibit significantly increased susceptibility to HSV-1 infection [12]. Although these studies were conducted in the context of HSV-1, they underscore the general importance of the cGAS/STING pathway in DNA virus defense. Our results indicate that cGAS/STING signaling activity, as reflected by the cGAMP-stimulated IFN-β response, is significantly reduced following PRV infection, suggesting that PRV suppresses type I interferon pathway activation. However, isoliquiritigenin treatment not only enhanced this cGAMP-mediated IFN-β response but also elevated the transcriptional levels of STING, TBK1, and IRF3. It is worth noting that some of the observed pathway effects may be indirect, resulting from reduced PRV replication. However, in the PRV+ISL group, the expression levels of cGAS and STAT1 were even higher than those in the Mock group (Figure 2A). This suggests that ISL may exert dual effects: inhibiting viral replication and positively modulating the cGAS/STING and JAK/STAT pathways. Nevertheless, our current data only support a correlation between ISL treatment and pathway activation. The direct molecular targets of ISL remain unclear. Future studies using binding assays and gene knockout experiments are needed to verify causality. In addition, more direct evidence is required to determine whether ISL directly interferes with PRV immune evasion.

PRV has evolved multiple strategies to antagonize this pathway. The envelope protein UL13 recruits the E3 ubiquitin ligase RNF5, promoting STING degradation through non-canonical K27/K29 ubiquitination [35]. Another viral protein, US2, facilitates K48-linked ubiquitination-dependent degradation of STING by recruiting the E3 ubiquitin ligase TRIM21 [36]. Our results show that isoliquiritigenin treatment reverses PRV-induced transcriptional suppression of STING, suggesting that it may counteract PRV immune evasion by maintaining STING expression. Furthermore, PRV infection significantly suppressed the mRNA expression of downstream signaling genes, including TBK1, IRF3, and IRF7, while isoliquiritigenin treatment effectively reversed this suppression, restoring their transcript levels to values significantly higher than those of the virus-only control. At the protein level, isoliquiritigenin also promoted IRF3 phosphorylation, with levels markedly rebounding and even becoming significantly enhanced following treatment. Collectively, these results suggest that isoliquiritigenin modulates the PRV-suppressed cGAS/STING pathway by upregulating STING, TBK1, and IRF3 mRNA expression, while also promoting IRF3 phosphorylation at the protein level, together contributing to counteracting PRV-mediated suppression of this pathway.

Beyond the cGAS/STING pathway, we also examined whether isoliquiritigenin affects downstream JAK/STAT signaling. Notably, exogenous expression of IFN-α or IFN-β in PK-15 cells has been shown to significantly inhibit PRV replication while maintaining cellular morphology and strongly inducing key effector molecules such as ISG15 [37]. In the present study, through dual validation in cellular and animal models, isoliquiritigenin treatment significantly enhanced ISG15 expression under PRV infection. This finding was consistent across both in vitro and in vivo settings, further supporting the role of isoliquiritigenin in activating the type I interferon pathway.

It has been established that PRV directly blocks downstream signaling cascades triggered by secreted type I interferons. The viral protein UL50, a deoxyuridine triphosphate hydrolase (dUTPase), targets interferon receptors by inducing IFNAR1 degradation via the lysosomal pathway, thereby directly blocking exogenous interferon signaling. This results in inhibition of STAT1 phosphorylation and subsequent ISG expression [38]. Furthermore, PRV potently inhibits JAK/STAT signaling by specifically interfering with JAK kinase or STAT protein functions through its encoded viral proteins [39]. Our findings are consistent with and extend these observations, demonstrating that isoliquiritigenin treatment significantly restores PRV-inhibited STAT1 phosphorylation, upregulates JAK1 and STAT1 mRNA expression, and promotes transcription of multiple key genes. To further investigate the potential role of STAT1 in the antiviral mechanism of isoliquiritigenin, we performed siRNA-mediated knockdown of STAT1 in Vero cells prior to isoliquiritigenin treatment and PRV infection. Vero cells are naturally deficient in type I interferon production, allowing us to effectively exclude the influence of upstream interferon signaling. As expected, STAT1 siRNA significantly reduced STAT1 protein expression compared with the blank control. Notably, even under STAT1-deficient conditions, isoliquiritigenin treatment still partially restored STAT1 phosphorylation in PRV-infected cells. This indicates that isoliquiritigenin can activate the JAK1/STAT pathway, thereby contributing to its antiviral efficacy against PRV. Collectively, these findings suggest that isoliquiritigenin may counteract PRV immune evasion through stimulation of the JAK/STAT signaling pathway.

To further verify whether isoliquiritigenin inhibits PRV proliferation by modulating the type I interferon response, a PRV-infected mouse model was established. Treatment with isoliquiritigenin improved clinical signs in infected mice, as evidenced by reduced incidence of morbidity (including lethargy, hunched posture, and labored breathing) compared to the PRV-only group, and effectively increased the survival rate. This mechanism is similar to that of rosmarinic acid, a natural compound that activates the cGAS/STING pathway, reverses PRV-induced inhibition, induces IFN-β expression, and thereby suppresses viral replication, alleviates inflammation, and reduces mortality both in vitro and in vivo [40]. Similarly, myricetin has been shown to impede PRV replication through the EGFR/PI3K/Akt and type I interferon signaling pathways [41,42,43]. These findings are consistent with our observations that isoliquiritigenin activates type I interferon signaling. The antiviral effects of both compounds are attributable to their capacity to enhance the host innate immune response and support the concept that “targeting host immune pathways” represents an effective strategy against PRV infection.

The present study further supports this notion, as isoliquiritigenin effectively reduces viral load in the heart, liver, spleen, lung, kidney, and brain tissues of PRV-infected mice while concurrently upregulating the transcriptional levels of genes associated with the cGAS/STING and JAK/STAT signaling pathways in these tissues. Furthermore, isoliquiritigenin enhanced p-IRF3 protein expression in brain and kidney tissues, indicating systemic activation of the cGAS/STING signaling pathway in mice. These findings suggest that isoliquiritigenin treatment may enhance host resistance to PRV at the tissue level, although the contribution of this pathway activation to tissue protection requires further investigation.

Several limitations of this study should be acknowledged. (1) An exogenous cGAMP positive control was not included in the cGAMP assay, so the IFN-β signal should be interpreted as an indirect functional readout. (2) STAT1 knockdown was performed in Vero cells rather than PK-15 cells, which are the natural host for PRV; Vero cells reduce endogenous interferon interference but do not fully mimic the natural infection microenvironment. (3) The evidence for cGAS/STING involvement remains correlative, and additional loss-of-function experiments such as STING or IRF3 knockdown/knockout are needed to provide definitive proof. (4) The 50 mg/kg dose was selected based on preliminary experiments, but pharmacokinetic and toxicity data for this dose are lacking. (5) Tissue molecular data were obtained from both moribund and surviving animals using random sampling; however, usable tissues were not available from all deceased animals, particularly in the PRV group, which had the highest mortality. Surviving animals in this group may represent a less severely affected subpopulation, but results from moribund animals were consistent with those from surviving groups, which partially mitigates the concern that the findings are solely driven by survivorship bias. (6) Although viral loads in brain tissues were reduced after ISL treatment, drug concentration in the brain was not measured, and it remains unclear whether ISL crosses the blood–brain barrier or acts indirectly through peripheral immunomodulation. (7) No obvious toxicity was observed in the ISL-only group, but histopathological analysis was not performed. (8) All in vivo efficacy data were obtained from mice; the PRV infection model in mice has been validated in multiple studies [44] and is generally accepted in the field, but validation in the natural host (pigs) is essential before translation to clinical or swine industry applications.

## 5. Conclusions

Notwithstanding the limitations discussed above, these findings suggest that isoliquiritigenin, a natural flavonoid, may inhibit PRV replication both in vitro and in vivo, in a manner associated with the cGAS/STING and JAK/STAT signaling pathways. This association was evidenced by elevated IFN-β mRNA levels, enhanced phosphorylation of IRF3 and STAT1, and upregulated transcription of key pathway genes, and was correlated with reduced viral loads in infected mice. Collectively, these results support the potential of isoliquiritigenin as a lead compound for the development of anti-PRV therapies.

## Figures and Tables

**Table 1:** Primer sequences used for real-time PCR.

Gene	Forward Primer Sequence (5′ → 3′)	Reverse Primer Sequence (5′ → 3′)
cGAS (pigs)	TTCTTTCACGTCTGTACCC	GCAGAAAATATGCCACAC
STING (pigs)	CCGCCTCATTGTCTACCAG	TGCCCATGGTAACCTCC
IRF3 (pigs)	AAGGTTGTCCCCATGTGTC	TGTACTGGTCGGAGGTGAG
IRF7 (pigs)	CCCTCTACCCCCATCTGTGA	GGTGTTAACCGCAGGTGAGA
IFN-β (pigs)	AGTGCATCCTCCAAATCGCT	GCTCATGGAAAGAGCTGTGGT
TBK1 (pigs)	CGCGGAAGACATAAGAAAACTGG	ATCCACTGGACGAAGGAAGC
JAK1 (pigs)	GTATGGCGGCATTCTCCAAA	TACTGCCCCTGAGCAAAGAG
STAT1 (pigs)	TCTGGCACAGTGGCTAGAAAATC	GAAAACGGATGGTGGCAAAC
IRF9 (pigs)	GTTACCACTGAGGCCCCTTT	GCAGCAGCGAGTAGTCTGAAT
OAS1 (pigs)	AAGAACTCCTTGGAGAGCTG	CCTGTATGTTGCACATCGTG
ISG15 (pigs)	GGGAGTATGACCTCAAGCCTA	ACACTCAATTTTGCCACAGC
β-actin (pigs)	GGACTTCGAGCAGGAGATGG	AGGAAGGAGGGCTGGAAGAG
cGAS (mouse)	GTCGGAGTTCAAAGGTGTGGA	GACTCAGCGGATTTCCTCGTG
STING (mouse)	TCGCACGAACTTGGACTACTG	CCAACTGAGGTATATGTCAGCAG
TBK1 (mouse)	ACTGGTGATCTCTATGCTGTCA	TTCTGGAAGTCCATACGCATTG
IRF3 (mouse)	CTGACAATAGCAAGGACCCTTA	AGGCCATCAAATAACTTCGGTA
IRF7 (mouse)	TGAGCGAAGAGAGCGAAGAG	CCAGTAGATCCAAGCTCCCG
IFN-β (mouse)	TCCGAGCAGAGATCTTCAGGAA	TGCAACCACCACTCATTCTGAG
JAK1 (mouse)	CTGTCTACTCCATGAGCCAGCT	CCTCATCCTTGTAGTCCAGCAG
STAT1 (mouse)	TGCCTATGATGTCTCGTTTG	TTGCTTTTCCGTATGTTGTG
IRF9 (mouse)	CATAGTTGGCACATGTGAGACA	ATCTCTCCAGCCGCTCTTAG
OAS1 (mouse)	GAGGTGGAGTTTGATGTGCTGC	GTGAAGCAGGTAGAGAACTCGC
ISG15 (mouse)	CATCCTGGTGAGGAACGAAAGG	CTCAGCCAGAACTGGTCTTCGT
MXI (mouse)	TGAAAGAAGGAGAGGAGTGG	CCTACCCCAGCAATGAAGT
β-actin (mouse)	GGCTGTATTCCCCTCCATCG	CCAGTTGGTAACAATGCCATGT

## Data Availability

The original contributions presented in this study are included in the article/Supplementary Materials. Further inquiries can be directed to the corresponding authors.

## Supplementary Materials

The following supporting information can be downloaded at: https://www.mdpi.com/article/10.3390/vetsci13090974/s1, the Supplementary Materials contain the raw data for all figures presented in this study, including the complete datasets for Figure 1, Figure 2, Figure 3 and Figure 4. Figure 5 and Figure A1 are derived from the same dataset, which is also included in the Supplementary Materials.

## Abbreviations

The following abbreviations are used in this manuscript:

CC_50_	half-maximal cytotoxic concentration
cGAMP	cyclic GMP-AMP
cGAS	cyclic GMP-AMP synthase
CPE	cytopathic effect
dpi	days post-infection
EMEM	Eagle’s Modified Essential Medium
FQ-PCR	fluorescent quantitative polymerase chain reaction
hpi	hours post-infection
IC_50_	half-maximal inhibitory concentration
IFN	interferon
IFNAR	interferon-α/β receptor
IRF3	interferon regulatory factor 3
IRF7	interferon regulatory factor 7
IRF9	interferon regulatory factor 9
ISG15	interferon-stimulated gene 15
ISL	isoliquiritigenin
JAK1	Janus kinase 1
MOI	multiplicity of infection
MX1	myxovirus resistance 1
OAS1	2′,5′-oligoadenylate synthetase 1
PBS	phosphate-buffered saline
PRR	pattern recognition receptor
PRV	pseudorabies virus
PVDF	polyvinylidene difluoride
SDS-PAGE	sodium dodecyl sulfate-polyacrylamide gel electrophoresis
STAT1	signal transducer and activator of transcription 1
STING	stimulator of interferon genes
TBK1	TANK-binding kinase 1
TCID_50_	median tissue culture infective dose
